# Advancing the science of dissemination and implementation: three "6th NIH Meetings" on training, measures, and methods

**DOI:** 10.1186/1748-5908-10-S1-A13

**Published:** 2015-08-20

**Authors:** Enola Proctor, Christopher Carpenter, C Hendricks Brown, Gila Neta, Russell Glasgow, Jeremy Grimshaw, Borsika Rabin, Maria Fernandez, Ross Brownson, Geoff Curran, Brian Mittmann, Linda Collins, Lawrence Palinkas, Naihua Duan, Andrea Wallace, Ken Wells, Rachel Tabak, Greg Aarons

**Affiliations:** 1 Washington University in St. Louis, St. Louis, MO 63130, USA; 2 Northwestern University, Chicago, IL 60611, USA; 3 National Cancer Institute, Rockville, MD 20850, USA; 4 University of Colorado, Boulder, CO, USA; 5 University of Ottowa, Ottawa, Ontario, Canada; 6 University of Texas, Austin, TX, USA; 7 VA / UAMS, Fayetteville, AK, USA; 8 VA Center for Implementation Practice and Research Support, Los Angeles, CA, USA; 9 Penn State, University Park, PA, USA; 10 University of Southern California, Los Angeles, CA, USA; 11 Columbia University, New York, NY, USA; 12 University of Iowa, Iowa City, IA, USA; 13 University of California, Los Angeles, Los Angeles, CA, USA; 14 University of California, San Diego, San Diego, CA, USA

## Introduction

Building on momentum in five NIH-supported meetings, "Advancing the Science of Dissemination and Implementation," the NIH convened three separate meetings during 2013-4, each addressing an overarching issue: "Developing a field-based approach to D&I research training"; "Measurement & Standardized Reporting"; and "Fit between Investigation and Research design?"

## Methods

Meeting themes were selected by the NIH D&I Workgroup, addressing pressing needs in the field and high-demand topics from prior meetings. Targeted groups of participants were invited to the meetings, each led by a team of researchers and NIH staff.

## Results

The training meeting yielded a map of current training, a field wide training vision, and a set of tensions--notably training for a rapidly evolving field. The research design workgroup produced a common terminology that crosses diverse fields of medicine and public health as well as disciplines, and categorized 27 different designs that have been used for dissemination and implementation research. The reporting workgroup identified four broad areas, planning, delivery, evaluation, and long-term outcomes as well as cross-cutting issues to provide reporting consistency. The meetings further generated a set of issues that cross-cut the three topics, including how to reflect the evolution of measurement, design and reporting within training programs and how to train reviewers, editors, decision-makers, and practitioners?

## Discussion

The three issue-focused meetings provided opportunity to take stock of the field ten years after the initial NIH D&I meeting and generated papers that both synthesize the stimulate important advances for the field.

## Developing dissemination and implementation reporting guidelines

Reporting guidelines improve the overall depth and quality of manuscripts across specialties and journals. No reporting guidelines for Dissemination and Implementation (D&I) research yet exist. We sought to develop a framework from which D&I reporting guidelines can be derived and to identify ideal metrics.

The National Institutes of Health held a series of invited state-of-the-science meetings to address key gaps and opportunities in D&I research in 2013. One of these focused on reporting and evaluation/measurement. This workgroup's objective was to identify key areas in need of better measurement and reporting at all stages of D&I research.

The workgroup concluded that the existing plethora of reporting guidelines mandated additional exploration before deriving D&I-specific reporting guidelines, and decided that a D&I framework divided into the planning, delivery, evaluation, and long-term outcome phases of research was an essential first-step.

The overriding objective of the D&I framework was to improve population health, health equity, social well-being, and health system efficiency. The workgroup identified the following stages to be essential: Planning: D&I intervention's evidence-basis and mechanism of change, setting characteristics like organizational capacity for change and resources, evaluability and scalability of the implementation strategy, key partnerships, and study design. Delivery: reach; adoption; implementation fidelity, dose, adaptation, and costs. Evaluation: primary outcome effectiveness including measured unintended consequences; explicit description of settings and intervention adaptation, including PRECIS criteria for pragmatism, and robustness of implementation effort. Long-term Outcomes: sustainability, evolvability, transportability; conditions under which the findings hold; and an economic evaluation. In addition, participants identified multiple cross-cutting elements extending across all stages, including: multilevel context; multiple stakeholder perspectives; and societal costs. The next step is to contrast existing reporting guidelines with this new D&I framework to highlight overlap or deficiencies relative to complete and consistent D&I research reporting, and to identify appropriate measurement instruments.

## An overview of research designs for dissemination and implementation

The fields of medicine and public health have made great progress in determining whether an intervention is efficacious or effective by conducting carefully crafted randomized clinical trials. In contrast to these designs to evaluate an intervention's efficacy or effectiveness, the designs for dissemination and implementation (D&I) research are not yet well established, a factor that has no doubt has impeded developing our knowledge of effective D&I. By its very nature D&I research is intimately connected to understanding how programs, practices, or policies work in different contexts, so there is more attention in D&I research on external validity, as contrasted to the heavy emphasis on internal validity that many of the randomized efficacy and some effectiveness trials address.

This presentation is a product of a workgroup meeting of 10 scientists and NIH staff, convened by NIH to facilitate D&I research. This committee addressed differences in terminology and provided a summary of the designs that have been used in D&I research, including both randomized and non-randomized studies. We identified 27 designs and found it useful to categorize these designs into several broad categories. One category of designs involves what can be termed the "traditional translational pipeline" of interventions that move step by step from efficacy, to effectiveness, to implementation research. A second major class of designs involves "hybrid designs," which combine elements of effectiveness and implementation research in one single design. Thirdly, we describe designs that are focused on quality improvement as the primary goal, in contrast to producing generalizable knowledge.

Several of these latter designs borrow from diverse areas of engineering.

We provide illustrations of these alternative designs and discuss cross-cutting issues, including community engagement and ethics in conducting implementation research.

## Advancing the science of dissemination and implementation: training, measures, and methods

Demand for training in the science of dissemination and implementation is high, reflected in oversubscribed registrations for the NIH meetings on Advancing the Science of D&I and applications to existing training programs. D&I training is provided in a small number of national programs, but given local university courses and degree programs and the growth of on-line webinars, a field-wide perspective on training is needed.

NIH convened a meeting of representative U.S. and Canadian trainers and trainees to in September 2013 to assess the field, identify cross-cutting themes, and develop a field-based training vision.

The training meeting yielded a map of current training, including NIH funded summer training institutes, a handful of Master's and PhD programs, individual graduate courses, CTSA Cores, and on line webinars. Program aims, participants and funding sources vary, as do training deliverables (content, skills, certificates, degrees, grant applications). Training gaps were identified, including programs at the doctoral level and those designed for decision-makers, and practitioners. Several serious challenges were identified, including: shaping and continually evolving training for a rapidly advancing field, establishing boundaries with related fields, targeting appropriate levels of training specificity versus generality, and sustaining high-intensity training. The meeting also generated issues that cross-cut with the measures/reporting and research design meetings. Participants underscored the importance of a regular national meeting to provide an intellectual home for those trained in D&I. Meeting products will include papers for publication reporting a field-based training vision.

The meeting yielded a map of current training in dissemination and implementation research as well as gaps and needs to be met through new training initiatives and a repository of training resources.

